# Performance Evaluation of the KRYPTOR Compact PLUS Analyzer-Based B.R.A.H.M.S. CgA Ⅱ KRYPTOR Assay for Chromogranin A Measurement

**DOI:** 10.3390/diagnostics11122400

**Published:** 2021-12-20

**Authors:** Yu Jeong Choi, Juhye Roh, Sinyoung Kim, Kyung-A Lee, Younhee Park

**Affiliations:** 1 Department of Laboratory Medicine, Severance Hospital, Yonsei University College of Medicine, Seoul 03722, Korea; yunk9275@yuhs.ac (Y.J.C.); sykim@yuhs.ac (S.K.); KAL1119@yuhs.ac (K.-A.L.); 2 Department of Laboratory Medicine, Hallym University Sacred Heart Hospital, Anyang 14068, Korea; jjroh@hallym.or.kr

**Keywords:** chromogranin A, neuroendocrine tumor, biomarker

## Abstract

Numerous immunoassays have been developed to measure the levels of chromogranin A (CgA), a useful biomarker for diagnosing and monitoring generally heterogeneous neuroendocrine tumors (NETs). Here, we evaluated the imprecision and linearity of three such assays: KRYPTOR (ThermoFisher Scientific), NEOLISA (EuroDiagnostica), and CgA-RIA (CisBio), using 123 samples for each assay. The correlation coefficients between the assays were 0.932 (CgA-RIA versus NEOLISA), 0.956 (KRYPTOR versus CgA-RIA), and 0.873 (NEOLISA versus KRYPTOR). KRYPTOR showed good precision, with percent coefficients of variation less than 5% for low and high concentration quality controls. Linearity was maintained over a wide concentration range. Comparison of CgA levels from three disease entities (NETs, non-NET pancreatic tumors, and prostate cancer) and healthy controls showed that patients with NETs had significantly higher CgA levels (*n* = 57, mean: 1.82 ± 0.43 log ng/mL) than healthy individuals (*n* = 20, mean: 1.51 ± 0.23 log ng/mL; *p* = 0.018). No other significant differences between groups were observed. All three immunoassays showed strong correlations in measured CgA levels. Because KRYPTOR operation uses a fully automated random-access system and requires shorter incubation times and smaller sample volumes, the KRYPTOR assay may improve laboratory workflow while maintaining satisfactory analytical performance.

## 1. Introduction

Chromogranin A (CgA) is a useful biomarker for diagnosis and monitoring of the extremely heterogeneous neuroendocrine tumors (NETs) [1]. NETs can arise in the stomach, intestines, rectum, pancreas, lungs, and adrenal and thyroid glands. One common feature of all NETs is that they originate from specialized cells belonging to the neuroendocrine (NE) system [2]. NE cells produce, mature, and exocytose secretory granules containing general neuroendocrine markers, such as CgA, and site-specific markers, such as amine and peptide hormones [3]. Although some NE cells are localized to a certain anatomic site and have precise functions (e.g., adrenal, pituitary, parathyroid), those belonging to the diffuse NE system make up a poorly defined thin layer dispersed throughout the bronchopulmonary and gastrointestinal systems [4]. Because NETs can arise in diverse anatomical sites and exhibit varying degrees of hormone secretion, diagnosis via tissue biopsy or imaging is limited. A specific amine or peptide hormone may serve as a biomarker for a specific type of NET. For example, gastrin may be used as a biomarker for gastrinoma, insulin for insulinoma, serotonin for small bowel NET, and glucagon for glucagonoma [4]. However, a more universal biomarker for all of these conditions is needed to facilitate early diagnosis. To date, CgA is the best available circulating biomarker for such applications. In addition, recent studies have suggested that CgA may also serve as a biomarker for cardiovascular disease, as well as diabetes [5,6].

CgA was the first of the eight members of the granin family to be identified. Granins are the major components of the soluble core of dense-core secretory granules in NE cells and are, therefore, secreted by these cells. Additionally, granins are thought to play important roles in granulogenesis, secretory protein sorting, and secretory granule maturation and condensation [7,8]. CgA is a heat-stable, hydrophilic protein composed of 439 amino acids. After translation, CgA undergoes post-translational processing to generate small peptides with specific biological activities, such as pancreastatin (corresponding to residues 250–301) and catestatin (corresponding to residues 352–372) [9]. CgA mRNAs and proteins are found in all types of neurons [10], indicating their potential as universal biomarkers.

Numerous immunoassays have been developed for measuring CgA levels. Among these, the B.R.A.H.M.S. CgA Ⅱ KRYPTOR assay (ThermoFisher Scientific, Waltham, MA, USA; KRYPTOR) uses time-resolved amplified cryptate emission (TRACE) technology, which allows for spectral and temporal distinction between signals of interest and unwanted background signals [11]. Moreover, the B.R.A.H.M.S. KRYPTOR Compact PLUS analyzer, which is the platform utilized for the assay, is a fully automated random-access system that requires less hands-on time and produces results more quickly.

In this study, we evaluated the analytical performance of KRYPTOR to determine whether it may improve workflow in laboratories. Three CgA assays were compared: KRYPTOR, NEOLISA Chromogranin A (EuroDiagnostica, Malmö, Sweden [NEOLISA]), and CgA-RIA CT (CisBio Codolet, France [CgA-RIA]). To assess the analytical performance of KRYPTOR, we evaluated its linearity, precision, and correlation with the other two CgA assays. We also compared measured CgA levels in samples from patients with three different disease entities (NETs, non-NET pancreatic tumors, and prostate cancer) to healthy individuals to determine whether serum CgA can be used to discriminate among these groups.

## 2. Materials and Methods

### 2.1. Sample Collection

Residual serum from 123 laboratory samples for which CgA levels had been determined by NEOLISA were collected and stored at −20 °C. Collected samples with known NEOLISA results were tested with KRYPTOR and CgA-RIA according to the manufacturer’s recommendations.

Research involving human specimens complied with all relevant national regulations, institutional policies, and the tenets of the Helsinki Declaration (as revised in 2013). The study was approved by the Institutional Review Board of Severance Hospital, Seoul, Korea (IRB no. 1-2020-0058). The requirement for informed consent was waived.

### 2.2. Linearity and Precision

To verify the linearity of the KRYPTOR assay, two samples with low and high concentrations were mixed to produce five concentrations in the following proportions: 4:0, 3:1, 2:2, 1:3, and 0:4. Each concentration was measured twice.

The low and high concentration quality control materials provided with the kit were measured five times on each day for five days, and the percent coefficient of variation (%CV) was calculated.

### 2.3. CgA Values Relative to Patient Diagnosis

To detect any significant differences in CgA values according to patient diagnosis, samples were divided into three disease groups: NETs, non-NET pancreatic tumors, and prostate cancer. Diffuse NETs, paragangliomas, pituitary tumors, adrenal gland tumors, Von Hippel–Lindau disease, and multiple endocrine neoplasia type 1 were included in the NET category. Samples not corresponding to any of the three groups were excluded. Patients taking proton pump inhibitors (PPIs) and samples with CgA values below the lower limit of detection were also excluded. Samples from the three disease groups were also compared with 20 additional samples collected from healthy control individuals.

### 2.4. Statistical Analysis

For statistical analysis, Microsoft Excel 2013 (Seattle, WA, USA), Analyse-it for Microsoft Excel Method Evaluation Edition version 5.40.2 (Analyse-it Software, Ltd., Leeds, UK), SPSS Statistics v.23 (SPSS Inc., Chicago, IL, USA), and Prism 8.0 (GraphPad Software Inc., La Jolla, CA, USA) were used. Passing–Bablok regression, Bland–Altman analysis, and Cohen’s kappa coefficient were used to evaluate correlation and agreement between the three assays. Linearity was evaluated by linear fit regression. The Mann–Whitney U test was used to evaluate significant differences in the distribution of CgA measurements depending on the patient’s diagnosis. Results were considered statistically significant when the *p*-value was less than 0.05.

## 3. Results

### 3.1. Patient Demographics

In total, 123 samples were collected from 118 patients including follow-up samples. Patient demographics are shown in Table 1. The mean age of the patients was 59.9 years (range: 17 to 90 years). The male to female ratio was 1.622:1. The most common diagnosis among the patients was prostate cancer (25.4%) followed by gastroenteropancreatic-NETs (19.5%). The NETs diagnosed in most patients were of pancreatic origin. Twenty-three (19.5%) patients were taking proton pump inhibitors (PPIs).

### 3.2. Assay Characteristics

The characteristics of the three different assays compared in this study are summarized in Table 2. All assays utilized two types of monoclonal antibodies with antigenic sites at different regions on CgA. NEOLISA and CgA-RIA operated in batch mode, while KRYPTOR operated in random-access mode. The required sample volume was 50 µL for NEOLISA and CgA-RIA but only 14 µL for KRYPTOR.

### 3.3. Correlation

#### 3.3.1. CgA-RIA versus NEOLISA

**Figure 1:**
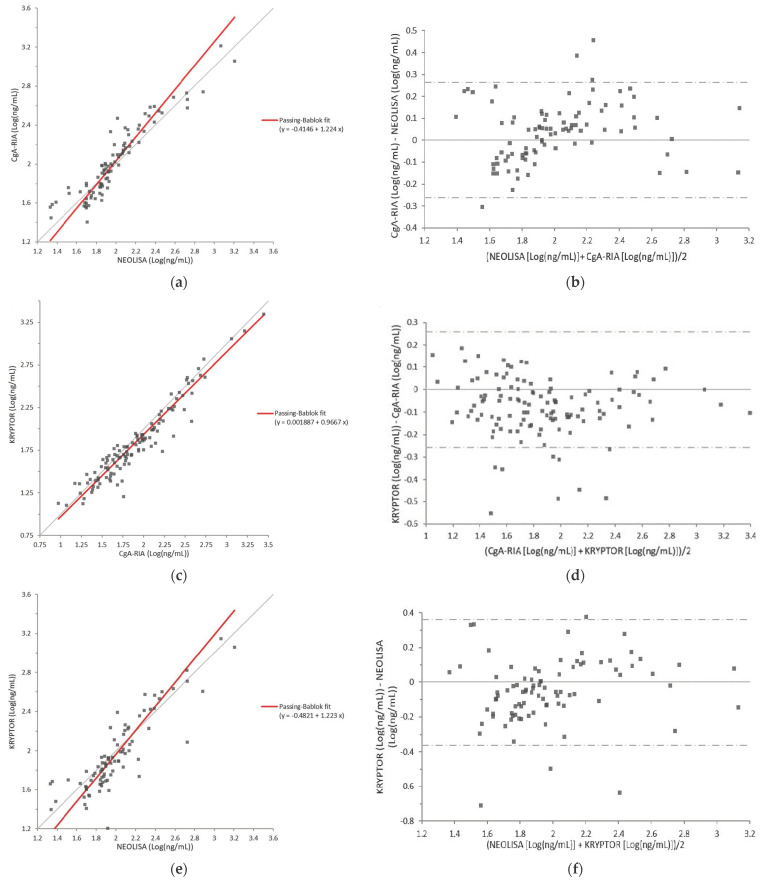
(**a**) Comparison between CgA-RIA and NEOLISA by Passing–Bablok analysis. The equation of the regression line was y = −0.415 + 1.224x, and correlation coefficient was 0.932. (**b**) Difference plot of CgA-RIA and NEOLISA measurements using Bland–Altman analysis. Average bias (0.036 log ng/mL; SD = 0.134 log ng/mL) is marked by a solid line, and the 95% confidence interval (CI) is shown by two dotted lines. (**c**) Comparison between KRYPTOR and CgA-RIA using Passing–Bablok analysis. The equation of the regression line was y = 0.002 + 0.967x, and the correlation coefficient was 0.956. (**d**) Difference plot of KRYPTOR and CgA-RIA measurements using Bland–Altman analysis. Average bias (−0.078 log ng/mL; SD = 0.132 log ng/mL) is marked by a solid line, and the 95% CI is shown by two dotted lines. (**e**) Comparison between KRYPTOR and NEOLISA using Passing–Bablok analysis. The equation of the regression line was y = −0.482 + 1.223x, and the correlation coefficient was 0.873. (**f**) Difference plot of KRYPTOR and NEOLISA measurements using Bland–Altman analysis. Average bias (−0.052 log ng/mL; SD = 0.185 log ng/mL) is marked by a solid line, and the 95% CI is shown by two dotted lines.

A comparison of the CgA-RIA and NEOLISA assays is shown in Figure 1a,b. Of the 123 samples collected, 90 were quantified using both assays. The rest could not be quantified because their CgA level was below the lower limit of quantification (LLOQ) of either CgA-RIA or NEOLISA. The results were compared using Passing–Bablok regression and Bland–Altman analysis. The slope of the regression line was 1.224 (95% confidence interval [CI]: 1.072–1.414) and the correlation coefficient was 0.932. The average bias between the methods was 0.036 log ng/mL (CgA-RIA—NEOLISA) with no specific trend as CgA levels increased.

#### 3.3.2. CgA-RIA versus KRYPTOR

A comparison of the CgA-RIA and KRYPTOR assays is shown in Figure 1c,d. Of the 123 samples collected, 118 were quantified by both assays. The results were compared using Passing–Bablok regression and Bland–Altman analysis. The slope of the regression line was 0.9667 (95% CI: 0.9054–1.024), and the correlation coefficient was 0.956. The average bias between the methods was −0.078 log ng/mL (KRYPTOR—CgA-RIA) with no specific trend as CgA levels increased.

#### 3.3.3. NEOLISA versus KRYPTOR

A comparison of the NEOLISA and KRYPTOR assays is shown in Figure 1e,f. Of the 123 samples collected, 118 were quantified by both assays. The results were compared using Passing–Bablok regression and Bland–Altman analysis. The slope of the regression line was 1.223 (95% CI: 1.055–1.408), and the correlation coefficient was 0.873. The average bias between the methods was −0.052 log ng/mL (NEOLISA—KRYPTOR) with no specific trend as CgA levels increased.

The categorical distribution of all samples according to the cut-off value of each assay is summarized in Table 3. Agreement between the qualitative results for CgA-RIA and KRYPTOR assays was 90.24% (111/123; κ: 0.763; 95% CI: 0.700–0.826). For KRYPTOR versus NEOLISA, the agreement was 91.06% (112/123; κ: 0.760; 95% CI: 0.692–0.828). For CgA-RIA versus NEOLISA, the agreement was 92.68% (114/123; κ: 0.826; 95% CI: 0.771–0.881). There were 16 discrepant samples in total. These samples measured a mean CgA value of 150.58 ng/mL (standard deviation [SD] = 53.23 ng/mL) using CgA-RIA with a cut-off of 98 ng/mL. The average values obtained by NEOLISA and KRYPTOR were 112.62 ng/mL (SD = 26.75 ng/mL) and 100.32 ng/mL (SD = 49.04 ng/mL), respectively.

### 3.4. Linearity

To validate the linearity of the KRYPTOR assay, five concentrations were prepared using quality control materials of low and high concentrations provided with the kit. Each sample was measured twice, and the average values are plotted in Supplementary Figure S1. The equation for the linear regression line was y = 0.9993x − 6.5174, and its determination coefficient was 0.9998.

### 3.5. Precision

The within-run %CVs for low (75.55 ng/mL; SD = 1.44 ng/mL) and high (486.6 ng/mL; SD = 10.46 ng/mL) concentration samples were 1.72% and 1.44%, respectively. For between-run %CVs, the values were 1.94% and 2.26% for the low and high concentration samples, respectively. All %CVs were within 5%, indicating good assay precision. The precision data are summarized in Table 4.

### 3.6. CgA Values According to Patient Diagnosis

**Figure 2:**
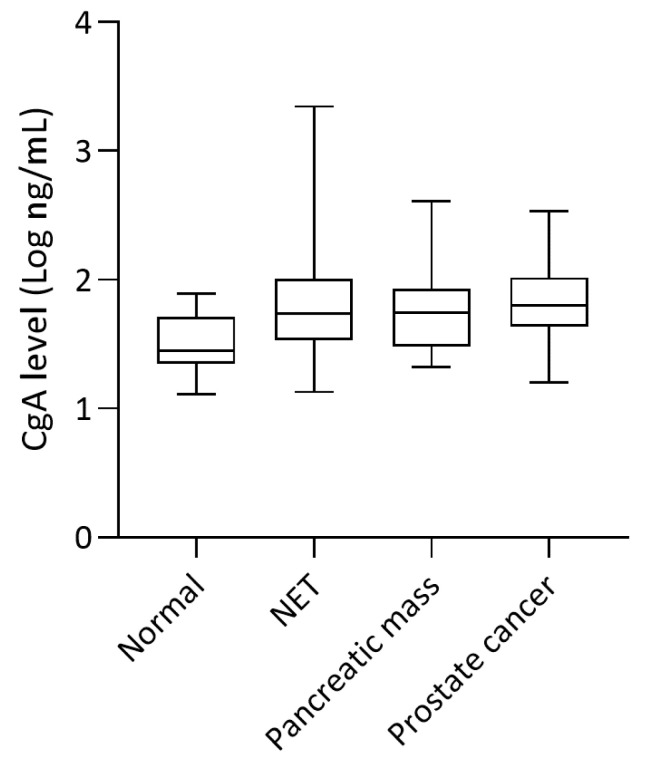
Box plot of CgA levels measured with KRYPTOR in relation to patient diagnosis.

CgA measured using KRYPTOR on samples from patients within the three study cohorts (NETs, non-NET pancreatic tumors, prostate cancer) and from healthy individuals were compared with the Mann–Whitney U test. Samples from patients taking PPIs and one sample from the NET group that measured below the lower limit of detection (11.8 ng/mL) were excluded. Median CgA levels in patients with NETs (*n* = 57), non-NET pancreatic tumors (*n* = 12), and prostate cancer (*n* = 16) were 1.74 log ng/mL (interquartile range (IQR): 0.87 log ng/mL), 1.75 log ng/mL (IQR: 0.44 log ng/mL), and 1.80 log ng/mL (IQR: 0.29 log ng/mL), respectively (Figure 2). For healthy individuals (*n* = 20), the median CgA level was 1.45 log ng/mL (IQR: 0.29 log ng/mL). The CgA levels in patients with NETs were significantly higher than those in healthy individuals (*p* = 0.018). However, there were no significant differences in any other comparisons.

## 4. Discussion

We compared three assays using three different methods: enzyme-linked immunosorbent assay (NEOLISA), TRACE (KRYPTOR), and radioimmunoassay (RIA; CgA-RIA) [11,16,17]. Overall, the analytical performance of KRYPTOR was acceptable. The assay was precise, with %CVs for the low and high concentration quality control materials below 5% for all conditions and showed linearity over a wide concentration range. Moreover, a comparison of the three assays demonstrated high correlation with each other (CgA-RIA versus NEOLISA, 0.932; KRYPTOR versus CgA-RIA, 0.956; and NEOLISA versus KRYPTOR, 0.873). The cut-off value provided for each assay was applied to examine the categorical distribution of the samples used in this study. Although the cut-off values provided for KRYPTOR and CgA-RIA were based on serum samples, those for NEOLISA was based on plasma samples. To verify whether the cut-off value for NEOLISA from plasma samples was also compatible with serum samples, CgA was measured using both plasma and serum samples from five patients with different CgA concentrations. Each sample was measured twice, and the average of the two values was plotted to obtain a linear regression equation of y = 1.264x − 0.8124 and determinant coefficient of 0.9817 (Supplementary Figure S2). Because the CgA levels in the serum and plasma samples measured by NEOLISA were strongly correlated, the cut-off value of NEOLISA for plasma samples was applied to the serum sample values obtained in our study. Our findings also showed substantial agreement among the three assays (Table 3). In total, 16 samples gave discrepant results in at least one of the assays. All discrepant samples tested above the cut-off value when measured with CgA-RIA, which was expected since CgA-RIA had the lowest cut-off value. Moreover, the average CgA levels of these 16 discrepant samples obtained using NEOLISA and KRYPTOR were much closer to the cut-off values for each assay, i.e., 108 ng/mL (NEOLISA) and 101.9 ng/mL (KRYPTOR). The demographic characteristics of these 16 patients exhibited no specific trend.

We found that CgA levels from patients with NETs were significantly higher than from healthy individuals, whereas those from patients with non-NET pancreatic tumors and prostate cancer were not significantly different from healthy individuals. When CgA levels from the three disease groups were compared, there were no significant differences. Studies reporting an association between CgA levels and tumor location observed the highest values in ileal NETs; pancreatic NETs exhibit intermediate values; and gastric, pituitary, and parathyroid tumors had even lower values than healthy individuals [18]. Therefore, the lack of significant differences may be explained by the fact that our NET group did not include samples from patients with ileal NETs but did include samples from 21 patients with pancreatic NETs and 20 patients with pituitary NETs. Moreover, although rare, prostate adenocarcinomas can undergo neuroendocrine differentiation during the later stages of disease progression [19]. In fact, elevated CgA levels in patients with prostate cancer has reportedly been correlated with the disease stage [20]. Patients with pancreatic adenocarcinomas have also been reported to show higher CgA levels than healthy individuals [21]. These factors may have contributed to the absence of significant differences in CgA levels for samples from patients with NETs, non-NET pancreatic tumors, and prostate cancer.

Our study had some limitations. First, the number of samples analyzed in this study was small. If samples were collected from a larger and more diverse patient cohort, their diagnoses may also have been more diverse. Thus, our analysis of CgA levels in patients with different types of NETs may have yielded significant differences between groups. Additionally, among the many factors affecting serum CgA concentrations, we only considered PPI administration. The most frequent causes of falsely (non-NET) elevated CgA levels in clinical practice are the use of PPIs, the presence of chronic atrophic gastritis, and impairment of kidney function [22,23]. Nevertheless, we did not consider patient factors other than PPI administration that could potentially influence CgA levels.

## 5. Conclusions

The overall analytical performance of the KRYPTOR assay was acceptable. Moreover, we observed a high correlation of KRYPTOR with other CgA assays. Because KRYPTOR operates using a fully automated random-access system and requires shorter incubation times and lower sample volumes, KRYPTOR assays may represent a superior workflow option with satisfactory analytical performance.

## Figures and Tables

**Table 1:** Patient demographics.

Variables	Patients (*n* = 118)
Age, years	
Mean (SD)	59.9 (17.1)
Sex, *n* (%)	
Male	73 (61.9)
Female	45 (38.1)
Main diagnosis, *n* (%)	
GEP-NET	
Pancreas	19 (16.1)
Liver	1 (0.8)
Stomach	1 (0.8)
Appendix	1 (0.8)
Rectum	1 (0.8)
Paraganglioma	1 (0.8)
Pituitary	
Tumor	19 (16.1)
Other	2 (1.7)
Thyroid disease	6 (5.1)
Adrenal gland tumor	12 (10.2)
Prostate cancer	30 (25.4)
Pancreatic tumor	14 (11.9)
Liver	
Hepatitis	1 (0.8)
HCC	1 (0.8)
Von Hippel–Lindau disease	2 (1.7)
MEN1	1 (0.8)
Other	6 (5.1)
PPI administration, *n* (%)	23 (19.5)

GEP-NET, gastroenteropancreatic neuroendocrine tumor; HCC, hepatocellular carcinoma; MEN1, multiple endocrine neoplasia type 1.

**Table 2:** Comparison of the three assays used in this study.

	Assay		
	NEOLISA Chromogranin A	B.R.A.H.M.S. CgA Ⅱ	CgA-RIA CT
Method	ELISA	TRACE *	RIA
Company name	EuroDiagnostica (Malmö, Sweden)	Thermo Fisher Scientific (Waltham, MA USA)	CisBio (Codolet, France)
Antibody	2 monoclonal	2 monoclonal	2 monoclonal
Epitope	Residues 236–251, 264–279 [12]	Residues 250–301, unknown [13]	Residues 145–245 [14]
Unit	ng/mL, nmol/L, U/L	ng/mL	ng/mL
Recommended specimen	Serum, EDTA/heparin plasma	Serum, EDTA plasma	Serum, plasma
Cut-off	Heparin plasma: ≤108 ng/mL (or 3.0 nM or 35 U/L)	Serum: <101.9 ng/mL	Serum: <98 ng/mL
Operating mode	Batch	Random-access	Batch
Incubation time	105 min	29 min	120 min
Required sample volume	50 µL	14 µL	50 µL

* TRACE: time-resolved amplified cryptate emission.

**Table 3:** Classification of CgA values measured with KRYPTOR, CgA-RIA, and NEOLISA.

CgA		Below Cut-Off	Above Cut-Off	Total	Cohen’s κ
		NEOLISA			
CgA-RIA	Below cut-off *	82	0	82	0.826 (95% CI: 0.771–0.881)
	Above cut-off *	9	32	41	
	Total	91	32	123	
		CgA-RIA			
KRYPTOR	Below cut-off †	82	12	94	0.763 (95% CI: 0.700–0.826)
	Above cut-off †	0	29	29	
	Total	82	41	123	
		KRYPTOR			
NEOLISA	Below cut-off ‡	87	4	91	0.760 (95% CI: 0.692–0.828)
	Above cut-off ‡	7	25	32	
	Total	94	29	123	

* Cut-off value of CgA-RIA CT: <98 ng/mL, † Cut-off value of KRYPTOR: <101.9 ng/mL, ‡ Cut-off value of NEOLISA: ≤108 ng/mL.

**Table 4:** Within-run, between-day, and total imprecision as recommended by Clinical and Laboratory Standard Institute (CLSI EP15-A3 [15]) *.

			Imprecision (%CV)		
Chromogranin A	Mean	SD	Within-Run	Between-Day	Total
Low	75.55	1.44	1.72	1.94	1.19
High	486.6	10.46	1.44	2.26	1.86

* In total, 25 replicates were tested at each concentration, including five replicates tested per day for five separate days. Concentrations are in ng/mL.

## Data Availability

All data generated or analyzed during this study are included in this published article and its Supplementary Information Files.

## Supplementary Materials

The following are available online at https://www.mdpi.com/article/10.3390/diagnostics11122400/s1, Figure S1: Linearity of KRYPTOR. An error rate of ± 10% is shown by the two dotted lines, Figure S2: Linear regression of average values of CgA levels measured using both plasma and serum samples from five patients having different concentrations of CgA.
