# Supplementary material for: Performance Evaluation of the KRYPTOR Compact PLUS Analyzer-Based B.R.A.H.M.S. CgA Ⅱ KRYPTOR Assay for Chromogranin A Measurement
Source: Diagnostics (Basel). 2021 Dec 20;11(12):2400. doi: 10.3390/diagnostics11122400 (PMC8700334; doi:10.3390/diagnostics11122400)
Supplement: Supplementary file 1 [file diagnostics-11-02400-s001.zip › diagnostics-1502482-supplementary.pdf]

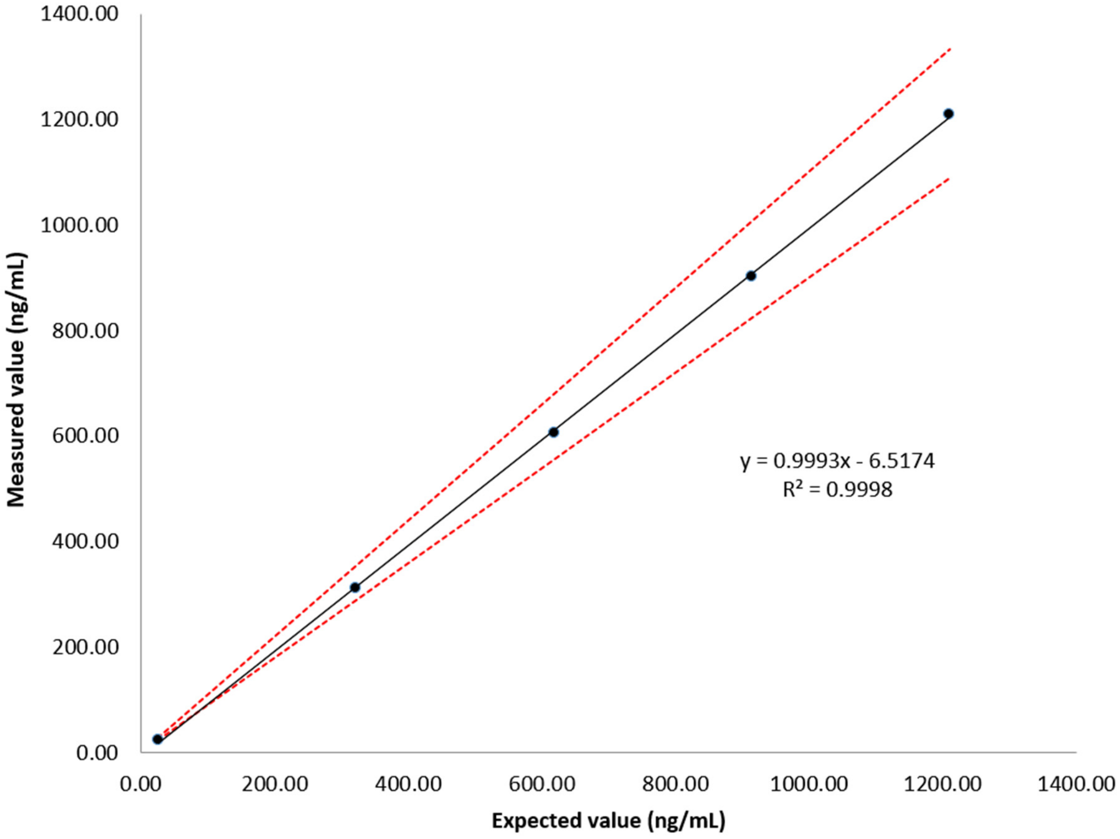

Figure S1. Linearity of KRYPTOR. An error rate of ± 10% is shown by the two dotted lines.

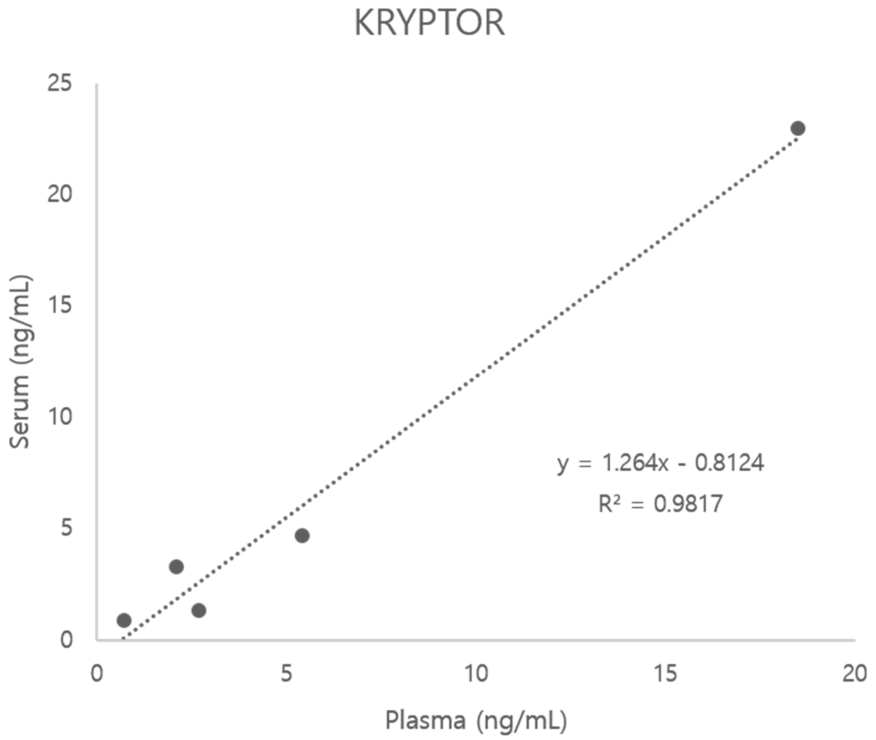

Figure S2. Linear regression of average values of CgA levels measured using both plasma and serum samples from five patients having different concentrations of CgA.
